# Cardiovascular and Functional Consequences of Lung Function Impairment in Northern Thai Agricultural Workers

**DOI:** 10.3390/ijerph22081168

**Published:** 2025-07-23

**Authors:** Anurak Wongta, Muhammad Samar, Nan Ei Moh Moh Kyi, Tipsuda Pintakham, Nootchakarn Sawarng, Surat Hongsibsong

**Affiliations:** 1 School of Health Sciences Research, Research Institute for Health Sciences, Chiang Mai University, Chiang Mai 50200, Thailand; muhammad_samar@cmu.ac.th (M.S.); naneimohmoh_kyi@cmu.ac.th (N.E.M.M.K.); tipsuda_pintakham@cmu.ac.th (T.P.); 2 Environmental, Occupational, and NCD Center of Excellent, Research Institute for Health Sciences, Chiang Mai University, Chiang Mai 50200, Thailand; 3 Department of Community Medicine, Faculty of Medicine, Chiang Mai University, Chiang Mai 50200, Thailand; nootchakarn_sawarng@cmu.ac.th; 4 Faculty of Public Health, Chiang Rai Rajabhat University, Chiang Rai 57100, Thailand

**Keywords:** Six-Minute Walk Test, lung function, cardiovascular response, agricultural health, spirometry, Thailand

## Abstract

In low- and middle-income countries, notably in rural agricultural populations exposed to environmental and occupational dangers, respiratory impairment and noncommunicable diseases (NCDs) are major public health issues. This cross-sectional study examined the associations between lung function, functional capacity, and cardiovascular responses to the Six-Minute Walk Test (6MWT) in 137 adults from San Pa Tong District, Northern Thailand. Lung function was assessed using spirometry, and participants were classified accordingly. Hemodynamic parameters, including blood pressure, heart rate, rate-pressure product, and oxygen saturation, were measured before and after the 6MWT. Participants with impaired lung function walked significantly shorter distances (*p* = 0.004), and walking distance was positively correlated with forced vital capacity (FVC) and forced expiratory volume in one second (FEV_1_). Logistic regression confirmed that walking distance independently predicted lung function impairment after adjusting for age and sex. Cardiovascular responses to exercise also varied significantly across demographic subgroups. These findings support the use of the 6MWT as a practical, cost-effective, and scalable method for detecting lung function impairments in resource-limited rural settings. To our knowledge, this is among the first studies to demonstrate the predictive value of the 6MWT for lung function impairment in a Southeast Asian agricultural population.

## 1. Introduction

Non-communicable diseases (NCDs), including cardiovascular and respiratory diseases, diabetes, and cancer, represent a growing global health issue, particularly in low- and middle-income countries (LMICs), where they impose considerable pressure on healthcare systems and economic development [1,2]. In Thailand, the burden of NCDs is increasing, disproportionately affecting rural populations, especially agricultural workers who experience limited access to healthcare, low health literacy, and chronic exposure to occupational and environmental hazards. Despite existing rural health initiatives such as the Village Health Volunteer (VHV) system and mobile screening units, these programs often overlook work-related risk factors. Agricultural communities face unique NCD risks from pesticide use, biomass smoke, and seasonal air pollution (e.g., PM2.5) [3,4,5,6], highlighting the need for integrated occupational and public health policies tailored to this vulnerable group.

Northern Thailand, which is a predominantly agricultural region, is particularly vulnerable to environmental and occupational health risks. Although agriculture is economically important, it exposes residents to chronic air pollution, particularly that of fine particulate matter (PM_2.5_). Agricultural burning, industrial discharge, and vehicular pollution were the main contributors to the increase in PM_2.5_. The heightened incidence of respiratory and cardiovascular disorders, along with higher mortality rates, has been linked to prolonged exposure to PM_2.5_ [7]. Although PM_2_._5_ exposure was not directly measured in this study, regional data were derived from government monitoring reports by the Thai Pollution Control Department and recent scientific studies documenting elevated PM_2_._5_ concentrations in Northern Thailand during agricultural burning seasons [8,9]. These findings highlight the urgent need for public health initiatives to reduce the persistent health effects of environmental contaminants.

Lung function impairment is a significant health concern in agricultural communities, mostly because of chronic exposure to air pollution and occupational hazards. In Northern Thailand, agricultural dust, insecticides, and biomass smoke are often absorbed by farmers, leading to respiratory irritation and reduced lung function [10,11]. These exposures increase the risk of chronic respiratory diseases, including chronic obstructive pulmonary disease (COPD), asthma, and hypersensitivity pneumonitis [12]. Additionally, pesticide exposure has been linked to rapid reduction in lung function [13]. Impaired lung function not only decreases physical capacity but also increases cardiovascular risk and lowers the overall quality of life, highlighting the need for effective occupational and public health interventions [14].

The Six-Minute Walk Test (6MWT) is a widely used, validated submaximal exercise test that measures the distance an individual can walk in six minutes [15,16]. It is a simple and reliable instrument for evaluating functional capacity, especially in individuals with chronic respiratory diseases, heart failure, and other disorders that affect their ability to exercise [17]. In addition to evaluating functional capacity, the 6MWT also offers insight into cardiovascular responses, such as changes in heart rate (HR), blood pressure (BP), and oxygen saturation (SatO_2_). Its ease of handling, cost-effectiveness, and low equipment requirements make it particularly useful in resource-limited environments, such as rural farming communities [14,18].

Most current research emphasizes health hazards and lifestyle variables instead of objectively evaluating exercise ability [5,19]. Although some studies have investigated respiratory health [4], few have used the 6MWT to evaluate functional capacity in individuals with impaired lung function. Understanding the influence of lung dysfunction on exercise performance and cardiovascular response is critical for identifying at-risk individuals, guiding targeted therapies, and establishing successful rehabilitation programs for this group.

This cross-sectional study sought to fill this information gap by comparing 6MWT performance, hemodynamic responses, and lung function status in adults with and without lung function impairment. This study hypothesized that impaired lung function is associated with reduced functional capacity and altered cardiovascular responses to submaximal exercise in rural agricultural workers. Unlike prior studies, this research uniquely examines this relationship using the 6MWT in a Southeast Asian low-resource agricultural context. The findings of this study are likely to provide useful insights into public health policies that focus on improving respiratory health and functional outcomes in this vulnerable group.

## 2. Materials and Methods

### 2.1. Study Design and Population

This cross-sectional study was performed from 30 October to 30 December 2023, in the San Pa Tong District of Chiang Mai, Thailand. The sample size was determined using G*Power 3.1, achieving 95% power, a Type I error rate of 0.05, and an effect size of 0.3. This effect size was chosen based on Cohen’s convention for a small-to-medium correlation in behavioral and health sciences, given the lack of prior data in similar rural populations [20]. The calculation yielded a required sample of 134 individuals. Inclusion criteria: adults ≥ 18 years old, in generally good health (self-reported), and able to perform the 6MWT. Exclusion: known asthma, COPD, orthopedic limitations, or cardiovascular disease under treatment. A total of 137 individuals were recruited by convenience sampling with support from local health offices and community leaders [21]. Most participants worked in small-scale subsistence farming typical of the Northern Thai region, including rice and maize cultivation, as well as seasonal vegetable farming. These activities commonly involve exposure to biomass smoke from burning crop residue, dust from soil preparation, and pesticide spraying. Such exposures represent well-documented respiratory and cardiovascular hazards in agricultural workers in Southeast Asia [5,8].

### 2.2. Ethical Approval and Consent

The study was approved by the Ethics Review Board of the Faculty of Associated Medical Technology, Chiang Mai University (approval no. AMSEC-66EX-062, approved on 26 October 2023 to 25 October 2024). All participants provided signed informed consent in accordance with the rules and regulations of the Declaration of Helsinki.

### 2.3. Data Collection Procedures

#### 2.3.1. Anthropometric and Hemodynamic Measurements

Anthropometric and hemodynamic measurements were conducted in the morning following standardized protocols. The participants wore light clothing and removed their shoes before the assessments. Weight and height were measured, and body mass index (BMI) was calculated as weight in kilograms divided by height squared in meters [22]. Waist circumference (WC) was assessed midway between the lowest rib and iliac crest with a non-elastic measuring tape. The waist-to-height and waist-to-hip ratios were then calculated. Resting brachial BP and HR were recorded in the sitting position using a validated oscillometric device (Omron Hem-8712, Omron Healthcare Co., Ltd., Kyoto, Japan). Post-exercise BP and HR were measured within one minute of completing the 6MWT. Rate pressure product (RPP) was calculated as systolic BP multiplied by HR, functioning as a recognized marker of cardiovascular stress [23]. Peripheral SatO_2_ was measured using a pulse oximeter before and after exercise.

#### 2.3.2. Spirometry Procedure

A SpiroScout spirometer (Ganshorn Medizin Electronic GmbH, Niederlauer, Germany) was used to perform spirometry according to the American Thoracic Society (ATS) guidelines [24]. All spirometry assessments were conducted by a certified technician from the Thoracic Society of Thailand under Royal Patronage (Certificate no 178/2566). The device was calibrated daily following ATS guidelines. The participants were instructed to avoid large meals, vigorous exercise, and smoking for at least two hours before testing. During the test, participants were seated with a nose clip and underwent the Forced Vital Capacity (FVC) procedure after receiving detailed instructions and demonstrations. Spirometry measurements included FVC, Forced Expiratory Volume in one second (FEV_1_), and the FEV_1_/FVC ratio. Each participant completed at least three acceptable maneuvers, ensuring artifact-free results with rapid starts and continuous exhalations. The highest values for FVC and FEV_1_ were recorded, depending on the difference between the largest and second-largest values being within 150 mL. Lung function impairment was defined as an FEV_1_/FVC ratio of <0.70 or an FEV_1_ % predicted value below 80% [24]. Spirometry results were used to classify the lung function patterns as normal or impaired, and impaired lung function was further categorized as restrictive or obstructive.

#### 2.3.3. Six-Minute Walk Test

The 6MWT was used to assess functional capacity in accordance with ATS standards. Participants were instructed to walk a standardized 30 m course for a duration of six minutes, with the total distance walked measured in meters. The 6MWT was conducted on a flat indoor 30 m concrete surface under ambient temperatures (25–28 °C), minimizing environmental variability. Following ATS guidelines, standard support was provided to all participants. Hemodynamic measures such as systolic blood pressure (SBP), diastolic blood pressure (DBP), HR, RPP, and SatO_2_ were assessed immediately before and after the test.

### 2.4. Data Analysis

Statistical analyses were performed using the Statistical Package for Social Sciences (SPSS) version 20. Descriptive statistics were used to summarize the participant characteristics and hemodynamic parameters. Continuous variables are presented as medians and interquartile ranges (IQR), and categorical variables are presented as frequencies and percentages. The Mann–Whitney U test was used to compare hemodynamic parameters and walking distance across groups. The Spearman correlation coefficient was used to assess the associations between post-6MWT hemodynamic measures, delta changes, walking distance, and spirometry outcomes. Logistic regression analysis was performed to investigate the relationship between lung function decline and walking distance after adjusting for age and sex. The cutoff value for statistical significance was set at *p* < 0.05.

## 3. Results

### 3.1. Demographic Characteristics and Hemodynamic Parameters

The study included 137 participants, most of whom were female (78.1%) and non-farm workers (73.7%). More than half of the participants (52.6%) were aged ≤ 60 years, and 92.7% had a normal body mass index (BMI). Lung function impairment was identified in 13.9% of participants. The median and IQR for hemodynamic parameters and walking distance, classified by demographic characteristics, are presented in Table 1 and Table 2.

Significant differences in hemodynamic parameters and walking distances were observed across demographic and clinical subgroups. Males exhibited significantly higher pre- and post-DBP than females (*p* < 0.001). Younger participants (≤60 years) also demonstrated higher pre-DBP (*p* = 0.007), post-DBP (*p* = 0.003), and walking distances (*p* = 0.004). Pre-HR (*p* = 0.006) and pre-RPP values (*p* = 0.036) were significantly lower in younger participants compared to those aged > 60 years.

Occupational variations were noticed in the post-DBP, with farm workers showing a slightly higher median post-DBP than non-farm workers (*p* = 0.034). Overweight/obese participants had significantly higher pre-SBP (*p* = 0.029), post-SBP (*p* = 0.012), and post-DBP (*p* = 0.036) values. However, their median walking distance was lower than that of participants with normal weight, although this difference was not statistically significant (*p* = 0.163).

Participants with impaired lung function exhibited a significantly lower median walking distance than those with normal lung function (*p* = 0.004). These findings highlight the variations in hemodynamic responses and functional capacity across different demographic and health-related characteristics, emphasizing the impact of lung function impairment on exercise tolerance.

### 3.2. Correlations Between Hemodynamic Parameters and Spirometry Results

Significant correlations were observed between spirometry results and hemodynamic responses following the 6MWT (Table 3). Walking distance demonstrated a positive correlation with FVC (r = 0.168, *p* < 0.05) and FEV_1_ (r = 0.219, *p* < 0.01), indicating that individuals with better lung function achieved longer walking distances during the test.

Post-DBP was positively correlated with FVC (r = 0.206, *p* < 0.05); however, z-score-adjusted DBP (z-DBP) revealed a more robust relationship with both FVC (r = 0.306, *p* < 0.01) and FEV_1_ (r = 0.252, *p* < 0.01). Delta hemodynamic measures, emphasizing the changes from pre- to post-6MWT, further underscored these associations. Delta PAS (variation in systolic blood pressure) had a significant connection with FVC (r = 0.213, *p* < 0.05) and FEV_1_ (r = 0.184, *p* < 0.05). Additionally, delta RPP (change in rate pressure product) was significantly correlated with FVC (r = 0.175, *p* < 0.05). No statistically significant correlations were detected between the other hemodynamic parameters and spirometry outcomes.

### 3.3. Logistic Regression Analysis of Lung Function and Walking Distance

The correlation between walking distance and lung function impairment was analyzed using logistic regression (Table 4). Variance inflation factors (VIFs) were below 1.1, indicating no multicollinearity. The Hosmer–Lemeshow test demonstrated sufficient model fit (*p* = 0.126). ROC analysis revealed an area under the curve (AUC) of 0.708 (95% CI: 0.601–0.816). The optimal cut-off for walking distance to predict lung function impairment was determined to be 415 m, yielding a sensitivity of 78% and specificity of 68%. The percent predicted values for FEV_1_ and FVC were calculated using the Siriraj reference equations for Thai adults [25]. Among the 19 participants with lung function impairment, 6 were classified as obstructive and 13 as restrictive patterns.

The optimal cut-off for walking distance to predict lung function impairment was determined to be 415 m, yielding a sensitivity of 78% and specificity of 68%. In the unadjusted model, walking distance was recognized as a significant predictor of lung function impairment, with an odds ratio (OR) of 0.993 (95% CI: 0.988–0.999, *p* < 0.05). This signifies that, for every extra meter walked, the likelihood of lung function impairment was reduced by 0.7%.

After controlling for sex and age, walking distance continued to be a significant independent predictor in the multivariable model, exhibiting an adjusted odds ratio (aOR) of 0.995 (95% CI: 0.989–1.000, *p* < 0.05). These data indicate that, irrespective of sex and age, increased walking distance correlates with a decreased probability of lung function impairment.

## 4. Discussion

This cross-sectional study investigated the functional capacity and cardiovascular responses to the 6MWT in individuals with and without lung function impairment in a rural agricultural community in Northern Thailand. The findings confirm that individuals with lung function impairment tend to walk shorter distances, highlighting the link between pulmonary function and exercise capacity. Furthermore, FVC and FEV_1_ exhibited a significant connection with walking distance, and logistic regression analysis validated the hypothesis that walking distance independently predicted lung function decline even after controlling for age and sex. Notable variations in hemodynamic parameters, especially diastolic blood pressure and heart rate, were detected across sex, age, obesity, and occupational categories.

### 4.1. Walking Distance and Respiratory Function

Individuals with lung function impairment and shorter walking distances are consistent with previous studies demonstrating the adverse effects of respiratory dysfunction on exercise capacity. Research related to COPD and interstitial lung disease (ILD) has consistently reported that lower lung function is associated with reduced 6MWT performance [26,27,28,29]. However, some studies indicate that 6MWT performance may also be influenced by other factors such as musculoskeletal fitness, motivation, and cardiovascular health [26,30], which suggests that walking distance should be interpreted cautiously when used as a proxy for lung function.

In our study, walking distance showed a positive correlation with FVC and FEV_1_, further supporting the link between pulmonary function and exercise performance. Individuals who walked longer distances had higher spirometry values, indicating greater lung capacity and airflow efficiency [31]. However, this relationship was modest, and previous studies have reported inconsistent correlations, particularly in populations with mixed restrictive and obstructive patterns [32].

These mixed findings may reflect differences in disease severity, test standardization, and participant characteristics. For instance, stronger associations are often reported in patients with advanced COPD or ILD, where exercise limitation is more pronounced, while our sample consisted of a rural community with generally mild or undiagnosed impairments. Additionally, protocol variations and comorbidities (e.g., orthopedic limitations) may further affect 6MWT outcomes [32,33]. These findings highlight the importance of interpreting the 6MWT within the context of the study population and clinical background.

Our results align with evidence from other LMIC regions, including India and Southeast Asia, linking occupational and environmental exposures, such as biomass smoke, to diminished lung function, with the Six-Minute Walk Test (6MWT) proving to be a viable screening tool for respiratory issues [34,35,36]. These similarities highlight the local significance of our findings and endorse the incorporation of functional evaluations like the 6MWT into health assessments within comparable rural communities.

### 4.2. Hemodynamic Responses and Clinical Implications

The hemodynamic responses observed in this study revealed significant physiological differences according to sex, age, and obesity status. Consistent with studies on sex-related differences in cardiovascular regulation, males showed higher DBP before and after the 6MWT than females [37]. Similarly, younger participants (≤60 years) demonstrated higher DBP and walking distance, aligning with age-related declines in cardiovascular and pulmonary function [30,38]. Although some subgroup differences in hemodynamic parameters were statistically significant, their clinical and pathophysiological relevance may be limited, particularly in asymptomatic individuals. Small variations in blood pressure or heart rate across subgroups may reflect normal physiological variability rather than indicators of disease risk [39,40].

The slightly higher post-exercise DBP observed in farmworkers may reflect greater physical stress and prolonged exposure to environmental pollutants associated with agricultural work; however, environmental exposure was not directly measured in this study and should be further investigated [41]. Furthermore, overweight/obese participants had higher pre- and post-SBP, pre- and post-DBP, and post-HR, but shorter walking distances, with no statistical significance (*p* = 0.163), which is similar to the findings of a previous study [42]. However, other studies suggest that some obese individuals may maintain preserved functional capacity, highlighting the importance of further studies on exercise responses in obesity [43].

### 4.3. Cardiovascular Adaptation to Exercise and Lung Function

The positive relationships between changes in SBP (delta-SBP) and RPP (delta-RPP) with FVC and FEV_1_ suggest that individuals with better lung function exhibit more significant cardiovascular responses to exercise. This indicates that higher pulmonary capacity facilitates more efficient cardiovascular adaptation during physical exertion. However, the sensitivity of delta-SBP and delta-RPP as indicators of cardiovascular adaptation has been questioned, particularly in individuals with mild lung function impairment. This is because these individuals may not exhibit significant cardiovascular responses [44]. This raises questions concerning the sensitivity of delta-SBP and delta-RPP as functional indicators, suggesting the need for additional cardiopulmonary assessments to comprehensively describe exercise responses in this population.

### 4.4. Limitations and Future Work

This study has several limitations. First, its cross-sectional design limits the causal inference regarding the link between lung function impairment and functional capacity. The use of convenience sampling may limit generalizability. However, recruitment was conducted with support from local health staff to ensure a diverse sample representative of the local community.

The findings may have limited generalizability beyond the study population. Although income data were not directly collected, socioeconomic vulnerability among rural workers likely contributes to the observed health disparities. Future studies should include income stratification to better assess the relationship between economic status and functional or respiratory outcomes.

This study also lacked direct quantification of occupational or environmental exposures. As such, conclusions about the impact of agricultural work on post-exercise hemodynamic responses should be interpreted cautiously. Additionally, BMI classification was excluded from analysis due to minimal variation and the lack of significant group differences. The absence of data on smoking status and undiagnosed respiratory conditions may limit comprehensive adjustment for confounders.

Finally, this analysis did not incorporate multivariable regression due to its exploration and the small number of participants with lung function impairment. Future research should implement multivariable models to better control for potential confounders and strengthen the validity of causal inferences.

Future research should use longitudinal designs to elucidate the temporal dynamics between environmental exposure and lung function impairment. Intervention studies are necessary to evaluate the efficacy of pulmonary rehabilitation, smoking cessation, and respiratory health education in agricultural workers. Future investigations should aim to identify and quantify the environmental and occupational factors that contribute to lung function impairment in these communities. Qualitative studies examining the experiences of individuals with lung function impairment could enhance their understanding of and inform patient-centered care. Broadening this investigation to diverse rural and agricultural settings, especially in LMICs, is essential for crafting focused public health strategies and regulations.

## 5. Conclusions

This study confirmed that persons with impaired lung function in a Northern Thai agricultural community showed reduced functional capacity, as indicated by shorter 6MWT distances. Walking distance may indicate functional limitations associated with lung function impairment, correlating with spirometry parameters (FVC and FEV_1_). Additionally, hemodynamic responses to the 6MWT varied by demographic factors, highlighting the complex interactions between lung function, cardiovascular adaptation, and functional capacity. To our knowledge, this is among the first studies to validate the predictive value of the 6MWT for lung function impairment in a rural Southeast Asian agricultural community. These results underscore the urgent need for targeted public health interventions to mitigate respiratory risks and functional limitations in agricultural communities affected by environmental and occupational exposures. The 6MWT is a useful, low-cost tool for evaluating and tracking functional capacity, especially in situations with limited resources. We recommend integrating portable spirometry devices and 6MWT into existing community health programs, such as those led by village health volunteers or mobile screening units, to enable the early detection of respiratory limitations in rural agricultural populations.

## Figures and Tables

**Table 1:** Demographical characteristics and hemodynamic parameters of the 6MWT (*N* = 137).

Characteristics		Gender			Age Range, Years			Occupation		
		Male	Female	*p*-Value	≤60	>60	*p*-Value	Farmworker	Non-Farmworker	*p*-Value
Frequency, *n* (%)		30 (21.9%)	107 (78.1%)		72 (52.6%)	65 (47.4%)		36 (26.3%)	101 (73.7%)	
Hemodynamic parameters, median (IQR)										
SBP, mmHg	Pre	134.5 (123.2–141.8)	132.0 (123.0–142.0)	0.668	132.0 (124.2–141.0)	132.0 (121.5–142.0)	0.947	135.5 (123.2–145.5)	130.0 (122.5–141.0)	0.222
	Post	140.5 (132.8–156.5)	133.0 (122.0–149.0)	0.070	135.0 (122.8–148.8)	133.0 (124.5–150.5)	0.955	136.0 (127.0–147.5)	134.0 (122.0–150.5)	0.466
DBP, mmHg	Pre	85.5 (79.0–92.2)	78.0 (71.0–85.0)	<0.001 **	81.0 (73.2–89.8)	77.0 (71.0–82.5)	0.007 **	80.5 (73.0–91.0)	79.0 (72.0–86.0)	0.239
	Post	87.5 (80.0–92.2)	78.0 (70.0–85.0)	<0.001 **	82.0 (74.0–90.8)	78.0 (68.0–84.0)	0.003 **	84.0 (74.0–89.5)	78.0 (71.0–86.5)	0.034
HR, bpm	Pre	79.0 (68.8–86.2)	77.0 (71.0–86.0)	0.898	74.0 (69.2–82.0)	81.0 (73.0–89.5)	0.006 **	77.0 (69.0–83.8)	77.0 (71.0–87.5)	0.523
	Post	84.0 (76.0–92.2)	83.0 (76.0–90.0)	0.932	83.0 (76.2–89.0)	83.0 (76.5–94.5)	0.486	83.5 (74.0–89.8)	83.0 (77.0–91.5)	0.739
RPP, mmHg x bpm	Pre	10,115 (9084–11,854)	10,285 (8960–11,390)	0.849	9885 (8684–11,082)	10,530 (9533–11,917)	0.036 *	10,392 (9034–11,826)	10,062 (8960–11,618)	0.625
	Post	11,450 (10,057–13,685)	10,944 (9462–12,780)	0.304	10,932 (9523–12,659)	11,424 (9420–13,373)	0.538	11,188 (10,022–13,275)	11,180 (9489–12,836)	0.854
SatO_2_	Pre	98.0 (96.0–99.0)	98.0 (96.0–99.0)	0.992	98.0 (97.0–99.0)	98.0 (96.0–99.0)	0.259	98.0 (96.0–99.0)	98.0 (97.0–99.0)	0.708
	Post	97.5 (96.0–98.2)	98.0 (96.0–99.0)	0.142	98.0 (97.0–99.0)	98.0 (96.0–99.0)	0.132	98.0 (97.0–99.0)	98.0 (96.0–99.0)	0.550
Walking distance, meters		444.5 (411.5–482.5)	424.0 (367.0–495.0)	0.368	450.0 (401.2–511.2)	412.0 (352.5–453.0)	0.004 **	416.0 (366.2–470.5)	440.0 (373.5–516.5)	0.120

Abbreviations: 6MWT, Six-Minute Walk Test; DBP, Diastolic Blood Pressure; HR, Heart Rate; RPP, Rate Pressure Product; SBP, Systolic Blood Pressure; SatO2, Saturation of Oxygen; IQR, Interquartile Range; * *p*-value < 0.05; ** *p*-value < 0.01 by Mann–Whitney U Test.

**Table 2:** Health status and hemodynamic parameters of the 6MWT (*N* = 137).

Characteristics		Obesity			Lung Function Impairment		
		Normal	Overweight/Obese	*p*-Value	Normal	Impaired	*p*-Value
Frequency, *n* (%)		127 (92.7%)	10 (7.3%)		118 (86.1%)	19 (13.9%)	
Hemodynamic parameters, median (IQR)							
SBP, mmHg	Pre	132.0 (122.0–141.0)	140.5 (130.8–153.5)	0.029 *	132.0 (123.0–141.0)	138.0 (121.0–146.0)	0.397
	Post	133.0 (122.0–148.0)	149.5 (143.0–159.0)	0.012 *	134.5 (123.5–149.2)	141.0 (125.0–157.0)	0.667
DBP, mmHg	Pre	79.0 (72.0–86.0)	88.5 (73.8–95.5)	0.057	79.0 (72.8–87.2)	74.0 (70.0–84.0)	0.116
	Post	79.0 (71.0–87.0)	87.0 (78.8–93.0)	0.036 *	80.0 (72.0–87.0)	73.0 (68.0–89.0)	0.405
HR, bpm	Pre	77.0 (70.0–86.0)	77.0 (72.5–79.2)	0.529	76.5 (70.0–86.0)	82.0 (74.0–91.0)	0.128
	Post	83.0 (76.0–90.0)	84.5 (78.0–94.5)	0.634	83.0 (76.0–89.0)	89.0 (78.0–100.0)	0.089
RPP, mmHg x bpm	Pre	10,140 (8946–11,620)	10,712 (9830–11,597)	0.325	10,071 (8936–11,617)	10,530 (9600–11,972)	0.190
	Post	11,160 (9460–12,825)	127,73 (10,848–13,891)	0.071	11,124 (9502–12,791)	11,808 (9568–14,500)	0.229
SatO_2_	Pre	98.0 (96.0–99.0)	97.5 (94.5–99.0)	0.817	98.0 (97.0–99.0)	97.0 (95.0–99.0)	0.322
	Post	98.0 (96.0–99.0)	99.0 (97.8–99.2)	0.055	98.0 (96.0–99.0)	98.0 (96.0–99.0)	0.654
Walking distance, meters		437.0 (375.0–495.0)	382.0 (350.0–452.5)	0.163	440.0 (383.8–507.8)	389.0 (326.0–420.0)	0.004 **

Abbreviations: 6MWT, Six-Minute Walk Test; DBP, Diastolic Blood Pressure; HR, Heart Rate; RPP, Rate Pressure Product; SBP, Systolic Blood Pressure; SatO2, Saturation of Oxygen; IQR, Interquartile Range; * *p*-value < 0.05; ** *p*-value < 0.01 by Mann–Whitney U Test.

**Table 3:** Correlations between hemodynamic parameters measured after the 6MWT and spirometry result.

Parameters	Spirometry Result		
	FVC	FEV1	FEV1%
Walking distance	0.168 *	0.219 **	0.071
Post-6MWT hemodynamic measurements			
SBP	0.064	0.115	0.020
z-SBP	0.064	0.115	0.020
DBP	0.206 *	0.155	−0.097
z-DBP	0.306 **	0.252 **	−0.162
HR	−0.031	−0.103	−0.077
RPP	0.006	0.000	−0.019
SatO_2_	−0.035	0.034	0.049
Delta (Post–Pre-6MWT) hemodynamic measurements			
Delta PAS	0.213 *	0.184 *	−0.013
Delta PAD	0.071	0.064	0.028
Delta HR	0.089	0.004	−0.071
Delta RPP	0.175 *	0.113	−0.055
Delta SatO_2_	−0.062	0.025	0.077

Abbreviations: 6MWT: Six-Minute Walk Test; SBP: Systolic Blood Pressure; DBP: Diastolic Blood Pressure; HR: Heart Rate; RPP: Rate Pressure Product; and SatO2: Saturation of Oxygen, * *p*-value < 0.05, ** *p*-value < 0.01.

**Table 4:** Logistic regression analysis of lung function and walking distance (N = 137).

Variable	Lung Function Impairment	
	OR (95% CI)	#aOR (95% CI)
Walking distance	0.993 (0.938–0.999) *	0.995 (0.989–1.000) *
Sex		1.415 (0.365–5.489)
Age		2.906(0.947–8.915)

Abbreviations: OR (odds ratio), * *p* < 0.05, by logistic regression analysis. # Adjusted for sex and age.

## Data Availability

The original contributions presented in the study are included in the article; further inquiries can be directed at the corresponding author.
